# A Biosynthetic Nerve Guide Conduit Based on Silk/SWNT/Fibronectin Nanocomposite for Peripheral Nerve Regeneration

**DOI:** 10.1371/journal.pone.0074417

**Published:** 2013-09-30

**Authors:** Fatemeh Mottaghitalab, Mehdi Farokhi, Arash Zaminy, Mehrdad Kokabi, Masoud Soleimani, Fereshteh Mirahmadi, Mohammad Ali Shokrgozar, Majid Sadeghizadeh

**Affiliations:** 1 Department of Nanobiotechnology, Faculty of Biological Sciences, Tarbiat Modares University, Tehran, Iran; 2 Department of Tissue Engineering, School of Advanced Technologies in Medicine, Tehran University of Medical Sciences, Tehran, Iran; 3 National Cell Bank of Iran, Pasteur Institute of Iran, Tehran, Iran; 4 Department of Polymer Engineering, Faculty of Chemical Engineering, Tarbiat Modares University, Tehran, Iran; 5 Department of Hematology, School of Medical Sciences, Tarbiat Modares University, Tehran, Iran; 6 Department of Genetics, Faculty of Biological Sciences, Tarbiat Modares University, Tehran, Iran; Osaka University, Japan

## Abstract

As a contribution to the functionality of nerve guide conduits (NGCs) in nerve tissue engineering, here we report a conduit processing technique through introduction and evaluation of topographical, physical and chemical cues. Porous structure of NGCs based on freeze-dried silk/single walled carbon nanotubes (SF/SWNTs) has shown a uniform chemical and physical structure with suitable electrical conductivity. Moreover, fibronectin (FN) containing nanofibers within the structure of SF/SWNT conduits produced through electrospinning process have shown aligned fashion with appropriate porosity and diameter. Moreover, fibronectin remained its bioactivity and influenced the adhesion and growth of U373 cell lines. The conduits were then implanted to 10 mm left sciatic nerve defects in rats. The histological assessment has shown that nerve regeneration has taken places in proximal region of implanted nerve after 5 weeks following surgery. Furthermore, nerve conduction velocities (NCV) and more myelinated axons were observed in SF/SWNT and SF/SWNT/FN groups after 5 weeks post implantation, indicating a functional recovery for the injured nerves. With immunohistochemistry, the higher S-100 expression of Schwann cells in SF/SWNT/FN conduits in comparison to other groups was confirmed. In conclusion, an oriented conduit of biocompatible SF/SWNT/FN has been fabricated with acceptable structure that is particularly applicable in nerve grafts.

## Introduction

Nowadays, a significant challenge in peripheral nerve regeneration is low recovery once injury [1], [2]. To date, several strategies have been exploited to mimic body's natural nerve regeneration process such as autologous nerve grafts, nerve transposition and nerve guide conduits (NGCs). Among them, applying of autologous nerve grafts is stressed. However, this method has some inevitable disadvantages including: extended surgery, donor nerve scarring, limited supply of donor nerves, sensitive neuroma formation and so on [3]–[5]. For these reasons, there has been motivation to develop alternative methods to treat peripheral nerve injuries not amenable to primary tensionless repair. Therefore, NGCs are developed because of their unavoidable advantages such as minimized suture line tension, increased concentration of endogenous proteins, and the presence of a selective barrier to permit the diffusion of nutritive molecules between the channel and the surrounding tissues [6]–[10]. A large number of materials have been tested to be used in NGCs, including laminin, collagen, poly-glycerol sebacate and polyglycolic acid (PGA) [11], [12]. However, some materials can bridge long gaps successfully and few conduits have the properties that may be expected of an “ideal NGC”. An idealized NGC must be biocompatible, biodegradable, soft and flexible, semipermeable, provide a guidance cue via tubular 3D structure, prevent fibrous tissue ingrowth and meet technical requirements for further production, sterilization, long-term storage, and surgical handling [1], [13]. For these reasons in the present study, silk/single walled carbon nanotube/fibronectin nanocomposites have been exploited to fabricate an ideal NGCs. Silk fibroin (SF) is a natural polymer with great biocompatibility, mechanical properties and suitable flexibility with the ability to be used in tissue engineering [14]. One of the important cues that should be considered in fabricating NGCs is the application of electrical conductive materials to support the electrical conduction of defected nerve during regeneration. While silk proteins have low electrical properties, single walled carbon nanotubes (SWNTs) were used in the present study in order to overcome this limitation. SWNTs are pure carbon atoms with high electrical conduction with the ability to interact with various polymers [15]–[18]. On the other hand, considering fiber orientation in nerve tissue scaffolding is mostly important. For this purpose, in the present work, aligned electrospun fibronectin (FN) nanofibers were incorporated in the structure of freeze-dried SF/SWNT substrates in order to simulate the elongated cellular pattern typical of native nerve tissue. Based on our knowledge, the application of aligned FN nanofibers in the structure of NGCs for nerve tissue engineering applications has been poorly investigated so far. It is considered that FN as a major multifunctional extracellular matrix glycoprotein could improve the biocompatibility of NGCs and mediates the behavior of Schwann cells and neurite extension through nerve regeneration process [19]–[21].

There are several techniques for NGCs fabrication such as freeze-drying, casting, self-assembly and electrospinning [22], [23]. In the present study, a novel hybrid method is introduced for NGCs fabrication based on freeze-drying and electrospinning methods. Firstly, SF/SWNT substrates were constructed using freeze-drying method. Secondly, aligned fibronectin nanofibers were electrospun on freeze-dried SF/SWNT and then rolled up to form a NGC.

Finally, this study aimed to prepare and comprehensively investigate the physical and chemical properties of SF/SWNT/FN based NGCs. Cells viability was also inspected in order to evaluate the ability of these scaffolds to support cell attachment and proliferation. To assess the efficacy of NGCs in repair of nerve sciatic, the *in vivo* study, histological analysis, electrophysiological assessment and immunohistochemistry were also performed.

## Materials

Sodium carbonate, lithium bromide (LiBr), 3500 Da cut off dialysis tube, formic acid, fibronectin (from human plasma, MW: 450 kDa), Dulbecco Modified Eagle's Medium (DMEM), fetal bovine serum (FBS), phosphate buffered saline (PBS), osmium tetroxide, [3-(4,5-dimethylthiazol-2-yl)-2,5-diphenyl tetrazolium bromide] (MTT), neutral red (NR), ketamine, xylazin, isopropanol, rabbit anti-mouse S-100 antibody (S2644) and Anti-Rabbit IgG/FITC (AF8035) were purchased from Sigma-Aldrich (USA). Carboxyl-Single-walled carbon nanotube (SWNT) was purchased from Neutrino Company of Iran. U373-MG cell line (Human glioblastoma-astrocytoma) was also obtained from National Cell Bank of Iran (NCBI).

## Methods

### Fabrication of SF/SWNT/FN NGC

**Figure 1:**
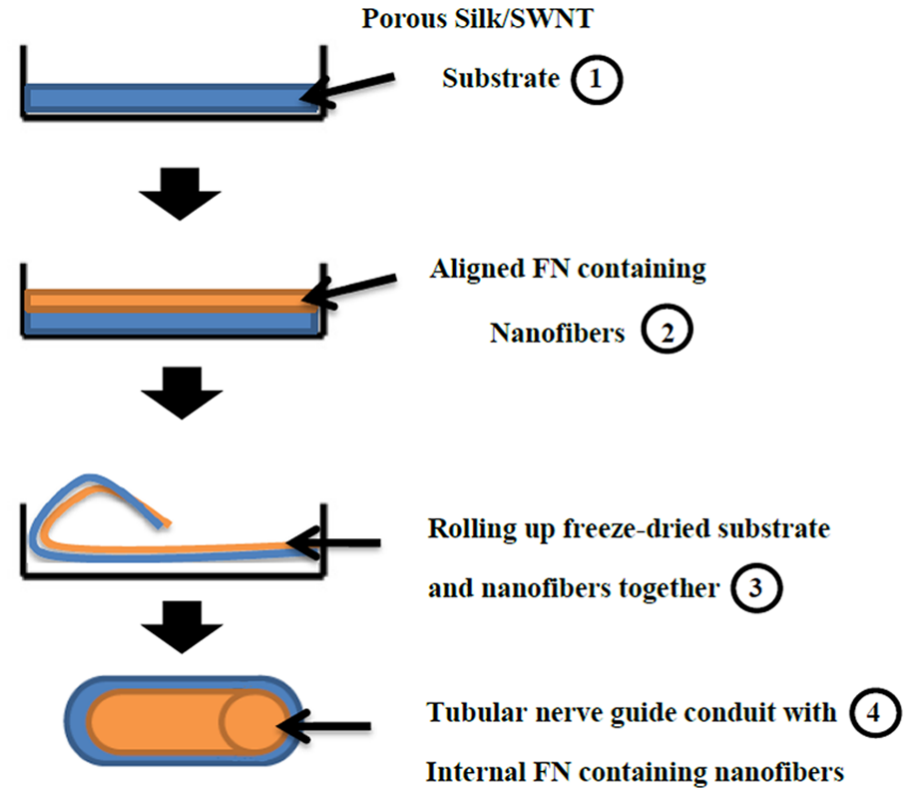
Schematic representation of rolling up protocol for NGC preparartion. 1) Porous SF/SWNT substrates prepared by freeze-drying. 2) Aligned FN containing nanofibers prepared by electrospinning on freeze-dried substrate. 3) Rolling up the complex together by manually manipulation of a flat-tweezer. 4) A tubular NGC with internal aligned nanofibers coated by porous Silk/SWNT substrate.

Firstly, Bombyx mori cocoons were degummed with 0.02% (w/v) Na_2_CO_3_ solution at 100°C for 60 min and then washed with distilled water. Degummed silk was dissolved in 9.3 M LiBr for 4 hours at 60°C and dialyzed in a cellulose tube against distilled water for 3 days at room temperature to gain 10% purified silk fibroin (SF) solution. Homogenizer apparatus (Germany) was used under 15000 rpm for 10 min in order to disperse SWNT in SF solution (0.2 mg/mL) uniformly. Finally, highly dispersed SF/SWNT solution was freeze-dried (OHRIST BETA 1–15, Germany) for 8 hours. It should be mentioned that the freeze-dried SF/SWNT was used as a substrate for further electrospinning process of aligned FN-containing nanofibers. To prepare the spinning solution, 0.13 gr of dried silk powders were dissolved in 1 mL of 98% formic acid (13% w/v) and stirred for 45 min. In addition, FN at a concentration of 0.5 μg/mL was dispersed in 13% SF solution to make a uniform solution suitable for electrospinning process. Characteristically, the electrospinnig process was performed under flow rate: 0.2, voltage: 30 kV, speed: 1500 rpm, and distance: 12 cm to prepare aligned FN-containing nanofibers on freeze-dried Silk/SWNT. Finally, the complex concerning nanofibers on freeze-dried substrate was rolled up together by manual manipulation with a flat-tip tweezers to prepare a tubular nerve guide conduit model. It should be mentioned that the complex was immersed in PBS for 4 hours before rolling up in order to increase its flexibility. The protocol of rolling up is presented schematically in figure 1.

### Characterization of NGCs structure

Field Emission Scanning electron microscopy ((FESEM; Quanta 200F, FEI, Oregon, U.S.) was performed in order to evaluate the structure of porous SF/SWNT substrates and aligned FN-containing nanofibers on the surface of SF/SWNT substrates.

To assess pore characteristics of aligned FN-containing nanofibers produced through electrospinning process, image processing analysis software was performed on high resolution SEM images. In this method, a binary image of the web was used as an input. Firstly, morphological reconstruction was used to identify the voids connected to the image border where the mask image was the input image and marker image was zero everywhere except along the border. The total area, which is the number of pixels in the image, was measured. Then the pores were labeled and each one was considered as an object. Here the number of pores may be obtained. Secondly, the number of pixels of each object as the area of that object was measured. Having the area of pores, the porosity and equivalent opening size (EOS) regarding to each pore may be calculated. The data in pixels may then be converted to nm. [24]. The following formula was used to measure pore size (O_i_):
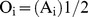
(1)


A_i_ is the diameter of a spherical particle that can pass the equivalent square opening. Furthermore, the pore size distribution (PSD) curve is plotted and O_50_, O_95_ and uniformity coefficient (Cu) are determined as below:
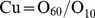
(2)Where O_X_ is an equivalent diameter determined from EOS. The ratio equals 1 for uniform openings and increases with decreasing uniformity of the openings.

Fiber diameter distribution was also measured by adjusted distance transform method applying on binary images obtained from SEM [25].

Attenuated total refractance (ATR) on a fourier transform infrared (FTIR; Germany) was implemented to assess the structural conformation of pure SF, SF/SWNT, and SF/SWNT/FN scaffolds in the range of 400–4000 cm^−1^.

Raman data were collected with Almega Instrument (Almega Thermo Nicolet Dispersive Raman Spectrometer; Germany), equipped with a CCD detector, and using a 785 nm laser diode as excitation source to examine the structural properties of SF prior and after addition of SWNT and FN. The spectral resolution of 4 cm^−1^ in the range of <3 cm^−1^ was also applied.

The thermal properties of SF prior and after methanol treatment and SF/SWNT structures were investigated using differential scanning calorimetry (DSC; NETZSCH, 200F3, Germany) at a rate of 10°C/min and in the temperature range of 30–350°C.

The electrical resistance of SF and SF/SWNT NGCs were tested using multi-meter (Keithley 2001, USA). Two probes were connected to NGCs via silver coated multifilament copper thread (22/20×0.04 mm, Elektrisola, China), which was wrapped around the tube. The samples were tested dry, at room temperature, at 1 cm intervals along its length.

The solution stability after dispersing carboxylated-SWNT in SF solution was inspected by measuring the backscattering of monochromatic light (λ = 880 nm) from the suspension using an optical analyzer [26].

### Fibronectin bioactivity

To determine the effects of electrospinnig parameters on the stability and biological activity of FN, a cell-based *in vitro* activity assay was performed. Firstly, U373 cells at the density of 5×10^4^ were seeded on FN (concentration: 0.5 μg/mL) loaded SF/SWNT conduits. The samples were incubated in DMEM at 37°C and 5% CO_2_ for 3, 7 and 14 days. As control group, equal concentration of FN was coated on SF/SWNT conduits by immersion method. The number of cells was then quantified using MTT assay. It should be noticed that the proliferation of control group was taken as 100% bioactivity.

### Biocompatibility

To quantify the number of viable cells next to NGCs, 2×10^5^ U373 cells were seeded on 24-well plate and incubated at 37°C with 5% CO_2_ for further MTT assay. After 24 hours, the NGCs were added to each well and remained for 3, 7 and 14 days. The cells were then stained with 2% MTT solution in PBS for 4 hours and the formed formazan was solubilized with isopropanol for 15 min. Absorbance was read at 570 nm. It should be mentioned that tissue culture polystyrene (TPS) and pure SF conduit were used as negative control and positive control groups, respectively.

Neutral Red (NR) assay was performed to evaluate the cellular adhesion on the NGCs. Concisely, 2×10^5^ U373cells/sample were seeded on the surface of NGCs and then the samples were incubated at 37°C with 5% CO_2_ for 3, 7 and 14 days. After that, the cells were washed with PBS and then 100 μL/well NR solution was added to each well. 4 hours later, NR solution was removed and then stain extraction solution (1% glacial acetic acid, 50% ethanol, and 49% distilled water) was added and re-incubated in 37°C and 5% CO_2_ for 15 minutes. The absorbance was measured at 570 nm using ELISA reader.

The cellular morphology on SF, SF/SWNT, and SF/SWNT/FN was examined by SEM. U373 cells at a density of 2×10^5^ were seeded on SF/SWNT/FN NGCs and were incubated at 37°C with 5% CO_2_. After 3 days, the samples were fixed in 1.5% glutaraldehyde solution for 4 hours. Afterward, they were dehydrated in graded alcohols concentrations; 10, 30, 50, 70, 80, 85, 90, 95, and 100%. A thin layer of Au/Pd was finally deposited on these substrates. Images were then acquired using a SEM at an accelerating voltage of 15 keV.

### *In vivo* study

#### Surgical procedure

All the procedures were approved by the local ethical committee of Pasteur institute of Iran. Fifteen adult Sprague–Dawley (SD) rats weighing 200–250 gr were used to evaluate the nerve regeneration. The animals were divided into 3 groups each with 5 rats. Group one: nerve defect group without any treatment as a negative control; group two: SF/SWNT conduits; group three: SF/SWNT/FN conduits. Animals were deeply anesthetized with a combination of ketamine (60 mg/kg) and xylazin (6 mg/kg) throughout the surgical procedure. Surgery was conducted on the rat's left leg, under aseptic condition. An operating microscope (Leica Micrisystems, Deerfield, IL) was used to divide the sciatic nerve near its origin, and a 10 mm nerve segment was excised with microscissors. The conduits were interposed into the 10 mm nerve defects. The proximal nerve was anchored in the conduit by 9–0 nylon microsutures. The distal end was then sutured into the other end of the conduit. Nerve stumps at both ends were sutured into the conduit to a length of approximately 1 mm.

#### Electrophysiological assessment

Electrophysiological evaluation was performed before sacrificing the animals for histological analysis. At first, all the animals were anesthetized with a combination of ketamine (60 mg/kg) and xylazin (6 mg/kg) and the left sciatic nerve were carefully exposed. Electrical stimuli were applied to the proximal portion of sciatic nerve trunk and recording needle electrode was placed in the anterior tibialis muscle. After that, nerve conduction velocity (NCV) was recorded. Nerve stimulation parameter used was 2 Hz pulses, 1 V strength, and 0.2 ms duration. In order to remove conduction of stimulation, the ground electrode was placed in surrounding muscle tissues.

#### Histological evaluation

Five weeks after implantation, all the animals were sacrificed by chloroform overdose. The implanted grafts were retrieve and histologically analyzed was performed as follow. The nerve grafts were immediately fixed in a cold buffered 3% glutaraldehyde solution for 48 hours. Nerve grafts were then washed in 0.1 M PBS. These segments were then dehydrated in a graded series of ethanol solutions, and finally embedded in paraffin. The embedded nerves were cut to 5 µm thickness, and then stained with hematoxylin/eosin (H&E) and toluidine blue. Moreover, the method described by Chang et al was used to quantify the axon regeneration [27]. Briefly, three randomly selected fields with an area of 80 µm×60 µm in each nerve specimen were observed at a magnification of 40x, the number and area of myelinated axons were counted. The numbers of axons were extrapolated by applying the area algorithm to estimate the total number of axons associated with each nerve. Analysis was conducted using an image analysis program (i-solution, IMT, Korea) from the captured light microscopy pictures.

#### Immunohistochemistry

The sections of the residual regenerated nerve tissue were used for S-100 immunohistochemistry analysis. After soaking in 0.01 M PBS for 10 min and blocked in a 5% normal goat serum for 60 min at room temperature, the nerve sections were incubated with rabbit anti-mouse S-100 antibody at 4°C for 24 h and subsequently reacted with the fluorescein isothiocyanate (FITC) labeled secondary antibody goat anti-rabbit IgG for 2 h at room temperature. The stained sections were observed under a fluorescent microscope (Zeiss, Germany).

#### Statistical analysis

All of the quantitative data were expressed as means ± standard deviation. Statistical comparisons were performed using one-way ANOVA with SPSS 16.0 (SPSS, USA). *P* values of less than 0.05 were considered statistically significant.

## Results and Discussion

### Characterization of NGCs structure

#### Scanning electron microscopy (SEM)

**Figure 2:**
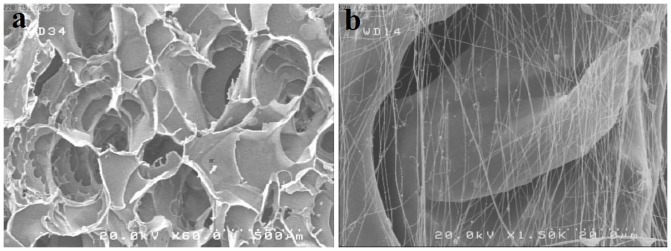
Scanning electron micrographs of: a) porous structure of freeze-died SF/SWNT conduits, b) Aligned fibronectin nanofibers produced through electrospining process.

**Figure 3:**
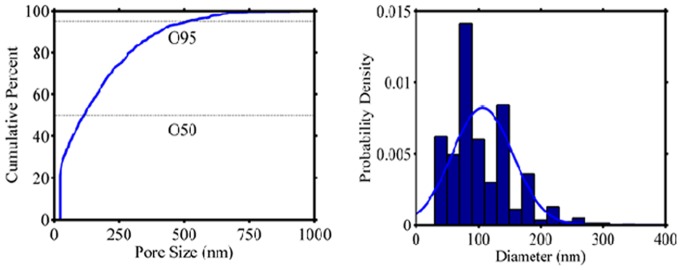
a) PSD curve of electrospun FN nanofibers, b) Diameter distribution histogram of FN nanofibers.

The morphology of conduits was evaluated using SEM. As shown in figure 2a, SF/SWNT conduits have shown highly porous structure (about 80%; data not shown) while figure 2b represents the aligned FN-containing nanofibers on freeze-dried SF/SWNT substrates with porosity and mean diameter of 50% and 106±46, respectively (figure 3a and 3b). It was considered that large pores of freeze-dried SF/SWNT substrates could prevent cellular spanning and cluster formation [28], [29]. On the other hand, SF/SWNT/FN conduits were found to support cellular adhesion and proliferation due to their appropriate pore size mimicking the structure of extracellular matrix (ECM). It is believed that these conduits with suitable porosity and oriented morphology could support nerve regeneration and also prevents the failure of defected nerve [27], [30]–[32].

#### FTIR

**Figure 4:**
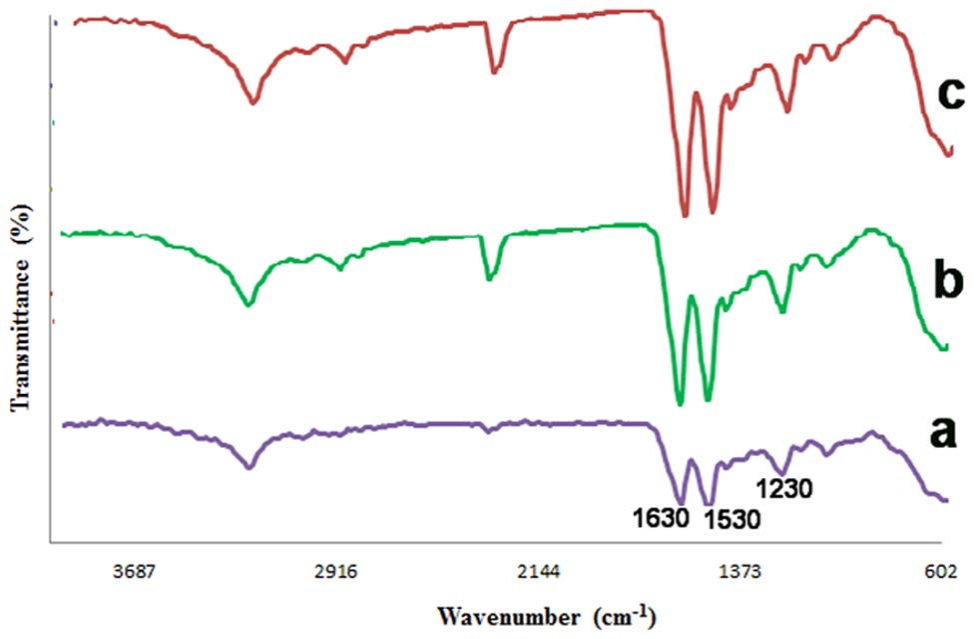
FTIR spectra of: a) Methanol treated pure SF. Three characteristic peaks in 1630 cm−1 (amide I), 1530 cm−1 (amide II), and 1230 cm−1 (amide III) are revealed due to β-sheet formation, b) SF/SWNT. The bands corresponding to amide I, II, and III are intensified to 1639−1; 1537 cm−1, and 1235 cm−1, c) SF/SWNT/FN. FN has also enhanced the typical peaks in comparison to SF/SWNT conduits.

It is illustrious that FTIR is an efficient method to recognize the structural changes of various macromolecules in different conditions. Figure 4 displays the FTIR spectrums of methanol treated pure SF, SF/SWNT and SF/SWNT/FN. The production of three characteristic peaks at 1630 cm^−1^ (amide I), 1530 cm^−1^ (amide II), and 1230 cm^−1^ (amide III) have indicated the formation of β-sheet in the secondary structure of pure silk fibroin due to methanol treatment (figure 4a) [33]. After the addition of carboxylated SWNT to SF, the bands corresponding to amide I, II, and III are intensified to 1639 cm^−1^, 1537 cm^−1^, and 1235 cm^−1^, respectively (figure 4b). It was considered that this might happen due to interaction between carboxylated SWNT and practical groups such as N-H in the structure of SF. Furthermore, an extra peak in 1410 cm^−1^ was reveled in SF/SWNT structure which might suggest the C-C interaction between SF and carboxylated SWNT. FN has also enhanced the typical peaks in SF/SWNT/FN conduits exceptionally in comparison to SF/SWNT conduits (figure 4c). It was suggested that adhesion of FN on SF/SWNT substrates might increase the β-sheet conformation in the backbone of the substrate and thus enhance the typical peaks corresponding to amid groups [26], [34].

#### Raman spectroscopy

**Figure 5:**
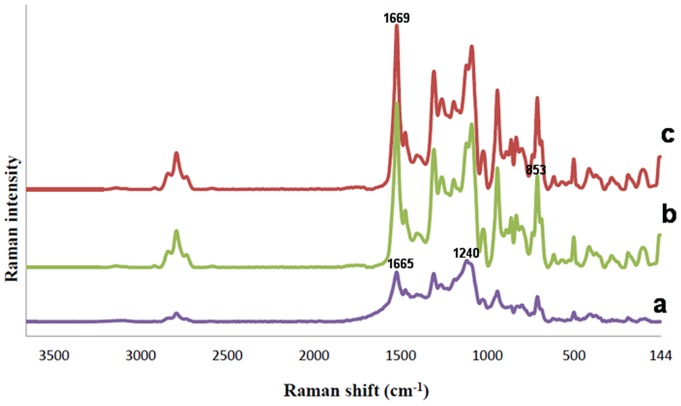
Raman spectra of: a) pure SF: the amide I band around 1665 cm−1, 1616 cm−1 for Trp, Phe and Tyr, 1240 cm−1 for amide III were observed, b) SF/SWNT: the peak correspondence to skeletal ν (C-C) associated in pure SF is removed, c) SF/SWNT/FN: the β-sheet structure in FN was identified by the characteristically amide I band around 1669 cm−1 and a higher intense band at 1245 cm−1 in the amide III region.

Creating protein-surface interactions is the primary step in several biological processes. Structure and orientation changes are often a part of interaction due to various factors such as homogen/non-hemogen dispersion, competitive adsorption/desorption processes, and experimental conditions [35]. Hence, in this study, Raman spectroscopy was performed to confirm the structural changes of SF prior and after the addition of SWNT and FN. Raman spectra of methanol treated SF substrates, as evidenced in table 1, clearly revealed their characteristic native secondary conformations. Briefly, the amide I band around 1665 cm−1, 1616 cm−1 for Trp, Phe and Tyr, 1240 cm−1 for amide III were observed (figure 5a). Additionally, in SF/SWNT substrates, the peak correspondence to skeletal ν (C-C) associated in pure SF was removed (at 1032 cm−1). Moreover, the vibration band related to Tyr was intensified to 853 cm−1 from 830 cm−1. It was observed that carboxylated SWNT could change the vibrational conformation in C-C bands associated in pure SF (figure 5b) [36]–[38]. In FN loaded SF/SWNT scaffolds, the β-sheet structure in FN was identified by the characteristically amide I band around 1669 cm−1 and a higher intense band at 1245 cm−1 in the amide III region (figure 5c). The FN molecules adsorbed on SF/SWNT surfaces were found to be modified in amide structure as compared to SF/SWNT structure due to surface binding.

**Table 1:** Main Raman band wavenumbers and assignments.

SF (cm^−1^)	SF/SWNT (cm^−1^)	SF/SWNT/FN (cm^−1^)	Assignment
2983	2936	2937	ν(C-H)
1660	1665	1669	Amide I
1616	1616	1616	Trp, Phe, Tyr
1452	1450	1448	δ(CH_2_) scissoring
1412	1412	1415	δ(C_α_H_2_)
1240	1240	1245	Amide III Silk I conf.
1176	1171	1173	ν(C-C)
1084	1086	1089	ν(C-C) skeletal, random coil, β-sheet
1032	(deleted)	Deleted	ν(C-C) skeletal
1003	1006	1004	Phe, Trp
830	853	832	Tyr
644	642	642	Amide IV

#### DSC

**Figure 6:**
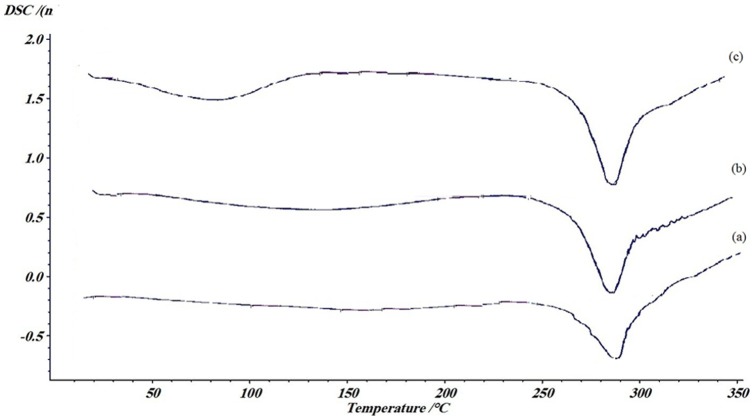
DSC thermogram of: a) pure SF, b) Pure SF after methanol treatment, c) SF/SWN substrates. Incorporating SWNT to the structure of methanol treated SF has a cumulative effect on growing the thermal stability to 288.4°C. A melting point absorption peak in the DSC trace of SF/SWNT in 77.7°C would also indicate the existence of SWNT.

Figure 6 demonstrates the DSC thermogram of SF before and after methanol treatment and addition of SWNT. Generally, SF displays two endothermic peaks, one below 100°C indicating loss of moisture and the other around 300°C representing crystallization [39]. It was reported in some studies that different treatment conditions could change these endothermic peaks relatively [40]. In the present study, the broad endothermic peak around 70°C was remained unchanged after treating SF with methanol (figure 6a and b). It is suggested that the remained water content in SF after applying drying condition may be responsible for this observation. The water content could widen the endothermic peak corresponding to dehydration [41]. Moreover, a little change (from 281.5 to 283.7°C, not significant) was observed in thermal decomposition peak in SF after methanol treatment (figure 6a and b). However, in contrast with our results, Motta et al. have reported that methanol treatment could induce the crystallization of SF [40]. This discrepancy may be due to applying different methodological procedures and treatment conditions. Additionally, incorporating SWNT to the structure of methanol treated SF has a cumulative effect on growing the dehydrating peak to 77.7°C and thermal decomposition peak to 288.4°C (figure 6c). This change may be attributed to uniform distribution of SWNT in SF solution. It is suggested that dispersion properties of SWNT in a solution could affect its thermal properties [42].

#### Solution stability

**Figure 7:**
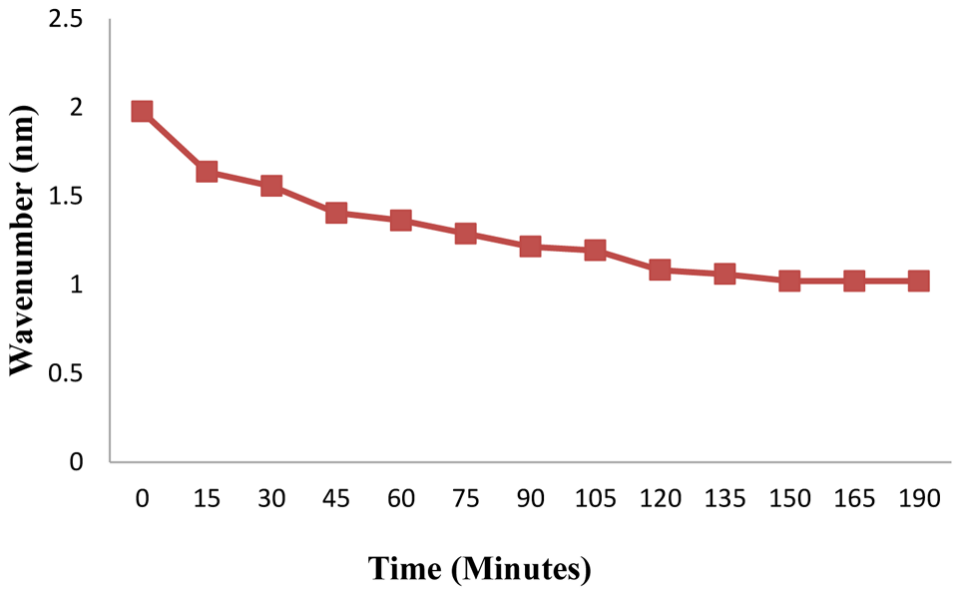
Solution stability curve of SWNT dispersion in SF solution. It seems that SF as an amphiphilic structure could act as a surfactant agent for dispersing carboxylated-SWNT in stabilized manner.

One of important parameters in fabricating scaffolds for tissue engineering applications is the stability of polymeric solutions before any processing procedures. Figure 7 presents a homogenous and stable SF/SWNT solution without sedimentation. Chemical modification processes of SWNT such as oxidation and carboxylation could induce the stabilization of the blend solutions [41], [42]. Therefore, carboxylated-SWNT was used in this study in order to form a stable solution of SF/SWNT. On the other hand, SF has a multi-block polymer architecture consisting of large hydrophobic and smaller hydrophilic internal blocks together with large hydrophilic chain end blocks [26]. Thus, it seems that SF as an amphiphilic structure could act as a surfactant agent for dispersing carboxylated-SWNT in stabilized manner.

#### Electrical conductivity

An ideal scaffold for peripheral nerve tissue engineering should have suitable electrical conductivity in order to support electrical transmission of injured nerve during regeneration [2], [4]. The electrical conductivity of SF/SWNT substrates were about 2.1×10^−3^ (S/m) while this was >10^−15^ (S/m) for pure SF (the upper limit of SF electrical conductivity is 10^−5^) [43]. It should be mentioned that several parameters could affect the electrical conduction of CNT reinforced composites such as polymer type and synthesis method, aspect ratio of CNTs, and uniform spatial distribution of individual CNTs [15]. For this purpose, in this study, SWNT with considerable electrical conduction has been used as a reinforcement molecule to enhance the electrical transmission of SF. In conclusion, it is considered that this study provides an appropriate SF/SWNT substrate to acquire enough electrical conductivity compared to natural nerve.

#### Fibronectin Bioactivity

**Figure 8:**
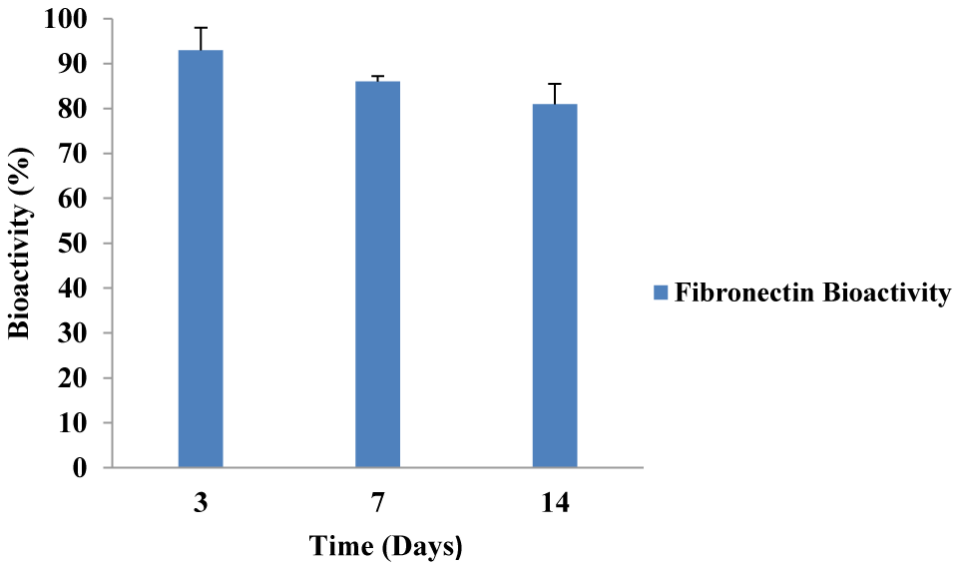
Fibronectin bioactivity after 14 days *in vitro* study. FN exhibited high bioactivity (about 85%).

The stability and bioactivity of electrospun FN is represented in figure 8. After 14 days of *in vitro* study, FN exhibited high bioactivity (about 85%) which could be attributed to very mild fabrication method for loading of FN on the scaffold. Similarly, Li et al. have shown that the bioactivity of BMP-2 was retained after the aqueous-based electrospinning process of silk fibroin fiber scaffolds containing BMP-2 [44].

#### Biocompatibility

**Figure 9:**
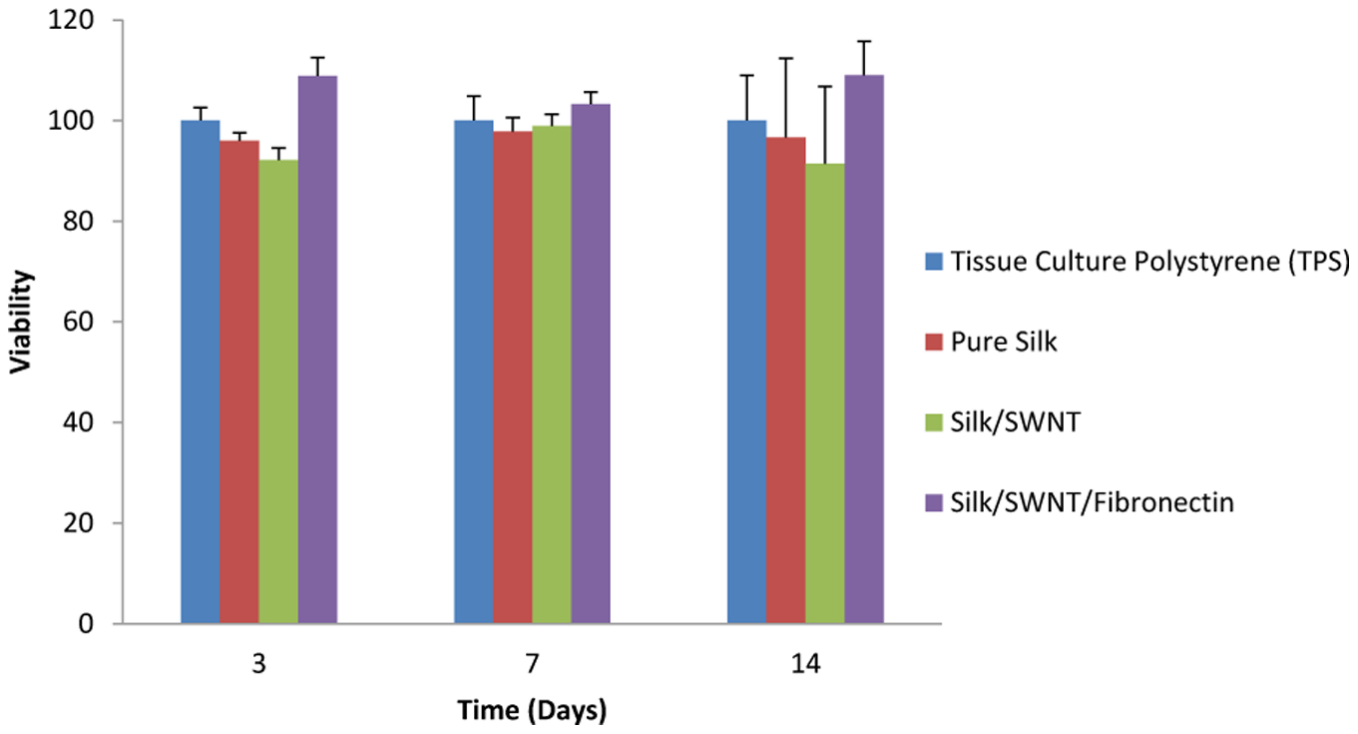
Viability of U373 cells (MTT assay). Less proliferation was observed for cells grown on SWNT containing conduits but not significantly.

**Figure 10:**
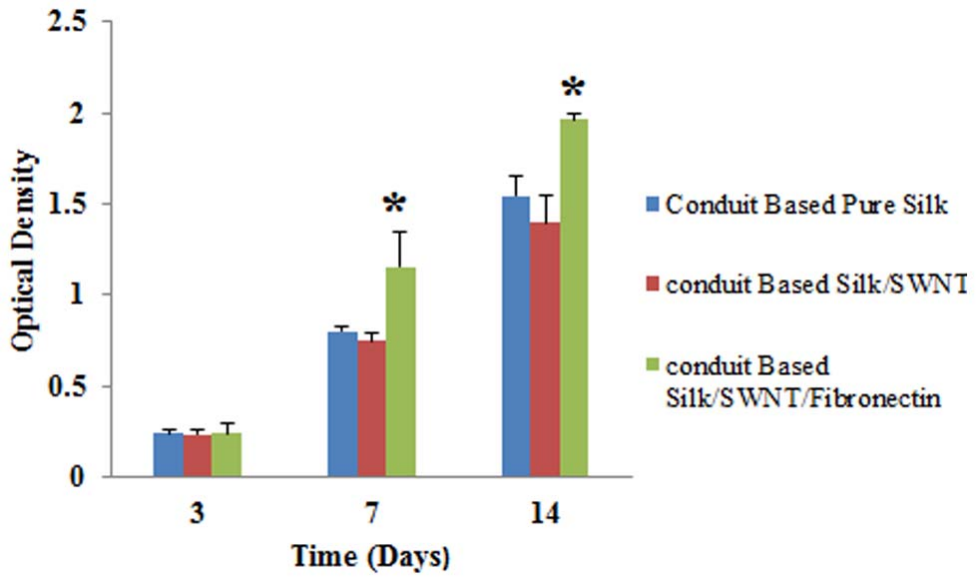
Attachment of the cells (NR assay). On day/SWNT/FN conduits was significant in comparison to all samples after 7^th^ and 14^th^ days. ^*^ *P*<0.05(compared with the pure SF and TPS).

The results presented in figure 9 showed the cells viability next to 3 different fabricated conduits. Cells viability was not statistically different compared with control group (TPS). Therefore, the fabrication processes in this study had no negative effect on cells viability. In addition, many studies reported some toxic effects of CNT on cells and tissues. For instance, stimulation of oxidative stress by CNTs could inhibit human keratinocytes proliferation, and provoked ultra-structural and morphological changes in these cells [45]. Although, in the present study, less proliferation was observed for cells grown on SWNT containing conduits but were not significant. This observation may result from: a) the harmful effects of SWNT is dose-dependent and for this, the concentration of SWNT applied in our study was very low (20 µg/ml) and also it is confirmed that the concentration of CNTs below 30 µg/ml did not have toxic effect on cells and tissues [46]. b) According to our solution stability result, SWNT was dispersed uniformly in SF solution and thus this issue could decrease the negative effects of SWNT on cultured cells. c) We used functionalized SWNT to fabricate conduits and it is generally agreed that functionalized CNTs have less adverse effects on cells [47]. The number of the cells adhered to fabricated conduits are shown in figure 10. The obtained results demonstrated that the cells proliferation on conduits could increase with incubation time. On day 3, there was not statistically different between all groups, but the proliferation of cells on SF/SWNT/FN conduits was significant in comparison to all samples after 7^th^ and 14^th^ days. This result indicated that FN loaded conduits could promote cells attachment and proliferation. The most important biological activity of FN is involved in cell migration, singling, adhesion and growth [48]. Similarly to our result, Dubey et al. reported that fibronectin-conjugated scaffolds had improved cell attachment and infiltration depth compared with scaffolds without and merely adsorbed fibronectin [49].

**Figure 11:**
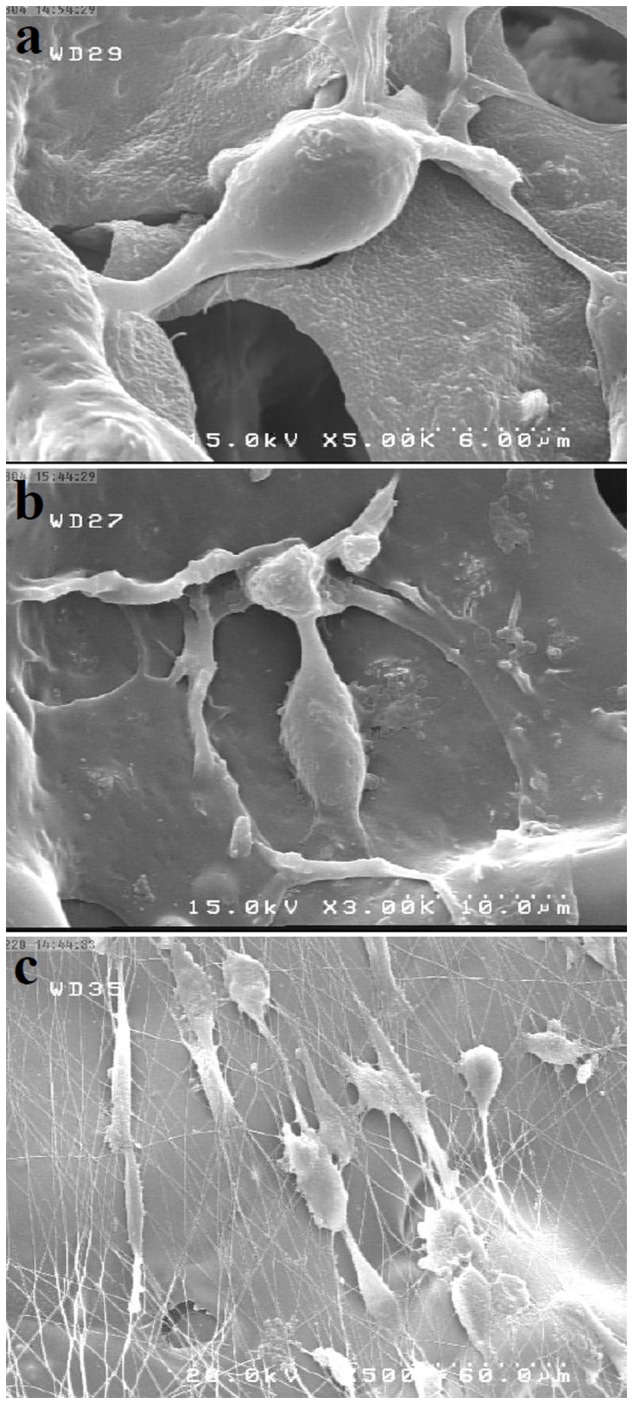
Scanning electron micrographs of U373 cells seeded on SF/SWNT/FN conduits. The cells were found to have attached and were distributed on aligned FN nanofibers with a spread out morphology.

The SEM images of cells grown on SF and SF/SWNT substrates had suggested that the proliferating cells could maintain their normal morphology while adhered on the surface of SF and SF/SWNT substrates (figure 11a and 11b). On the other hand, the cells grown on SF/SWNT/FN were found to have attached and distributed on aligned FN nanofibers with a spread out morphology (figure 11c). It is well-known that oriented morphology of nanofibers containing conduits could promote cellular direction while adhered to the scaffold and influence their functionality [50]. In consistent with previous studies, it was observed that the number of cells attached on the surface of SF/SWNT/FN in higher than SF and SF/SWNT substrates [19]. Finally, the fabricated conduits in this study were demonstrated to support normal growth of neural cells.

### *In vivo* study

#### Electrophysiological assessment

**Figure 12:**
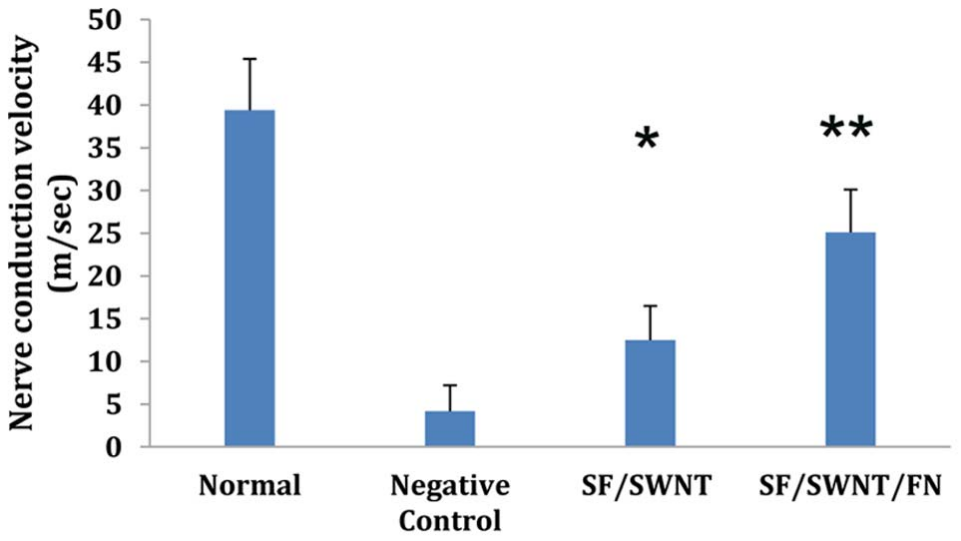
Nerve conduction velocities (NCVs) of normal sciatic nerve, negative control, SF/SWNT and SF/SWNT/FN after 5 weeks implantation in rats compared with each other (n = 3). *P<0.05 in comparison to negative control group. **P<0.05 in comparison to negative control and SF/SWNT groups.

In order to determine whether the functional reinnervation happened through the fabricated conduits or not, the electrophysiological analysis was accomplished. NCV was observed in SF/SWNT and SF/SWNT/FN groups after 5 weeks post implantation, indicating a functional recovery for the injured nerves (figure 12). However, NCV of the normal sciatic nerve was significantly higher (*p*<0.05) than that of the regenerated nerve in all groups (39.4±3 m/s). In nerve defect group without any treatment (negative control group) only weak signals were detected in location where the natural nerve conduit was removed except the formation of white fibrous tissue around the thighbone of the operated rats (figure 12). The diameter of axons, the thickness of myelin sheath and the length of internodes are three important factors that can influence the conduction velocity [51]. Regenerated nerves are often smaller in axon diameter and thinner in myelin sheath than normal nerves and for this; it is therefore expected that the conduction velocity in a regenerated nerve is lower than that of a normal nerve [5]. The NCV of the SF/SWNT/FN conduit was faster than that of the SF/SWNT conduit, which is statistically significant. This may be owing to the incorporation of FN in NGCs which could support nerve regeneration more than SF/SWNT group.

#### Histological assessment

**Figure 13:**
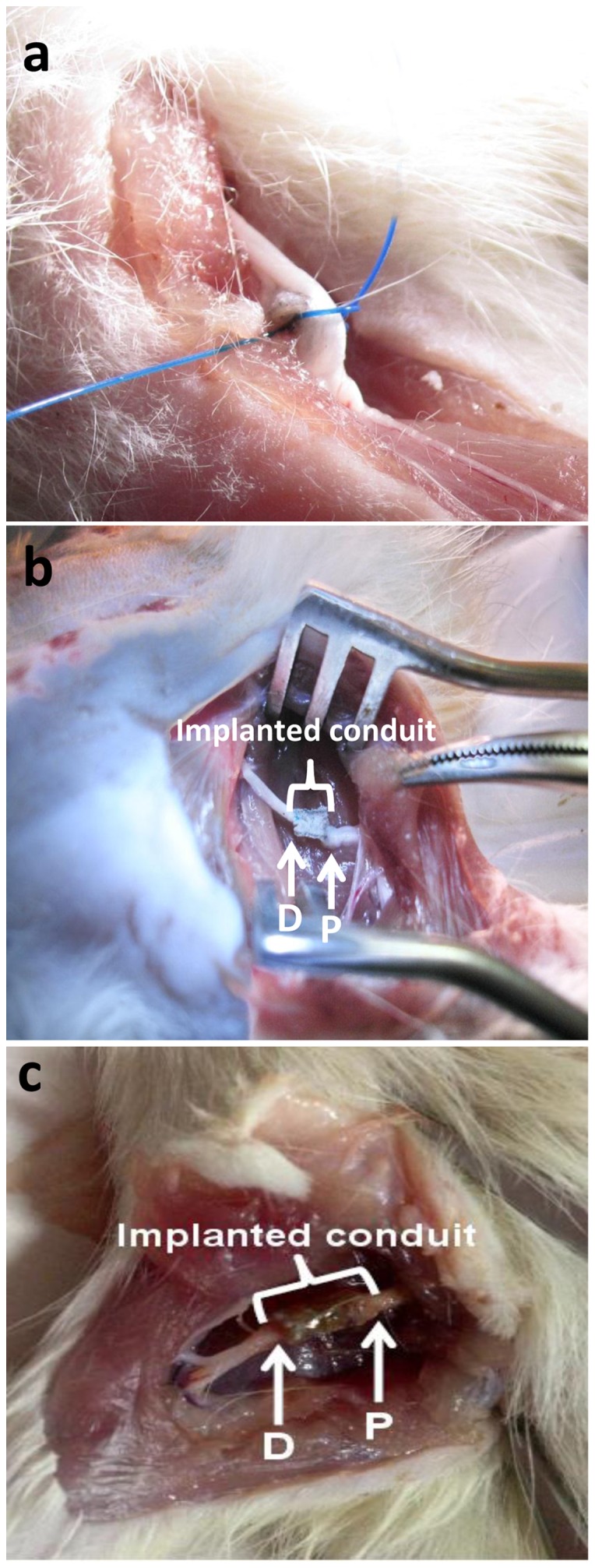
Inclusive observation after 5 a) Defected sciatic nerve without NGC (control group), b) Implanted SF/SWNT conduit, c) Implanted SF/SWNT/FN conduit.

**Figure 14:**
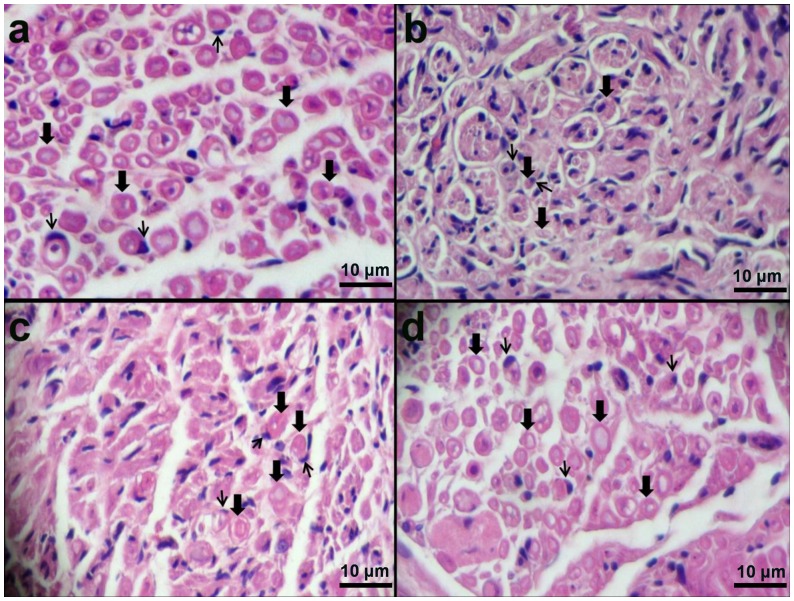
Histological assessment of NGCs after 5 a) Normal nerve, b) negative control (Defected sciatic nerve without NGC), c) Defects filled by SF/SWNT conduits, d) Defects filled by SF/SWNT/FN conduits. **Wide** **arrows**: myelinated axon. **Narrow** **arrows**: Schwann cells.

**Figure 15:**
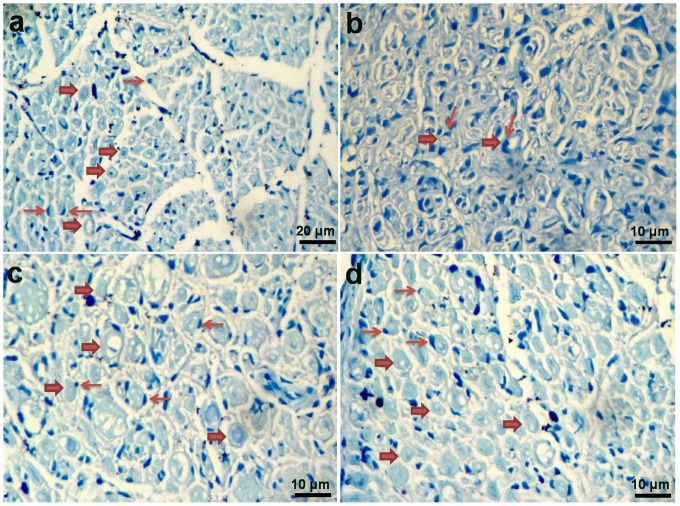
Cross sections of regenerated nerves taken from types of nerve conduits implanted in rats for 5 a) Normal nerve, b) negative control (Defected sciatic nerve without NGC), c) Defects filled by SF/SWNT conduits, d) Defects filled by SF/SWNT/FN conduits. **Wide arrows**: myelinated axon. **Narrow arrows**: Schwann cells.

After 5 weeks implantation, the structure of fabricated NGCs was stable as an important factor for axon regeneration and growth (figure 13a–c). A thin capsule of fibrous tissue grew and covered the implanted NGCs. After opening the conduits, the nerve was successfully connected and bridged; a newly regenerated nerve showed through the conduits (data not shown). It should be mentioned that in negative control group, no noticeable axon regeneration has been observed. Although, in SF/SWNT/FN conduits, numerous bundles of regenerated sciatic nerve fibers were clearly found in the proximal sections of the regenerated tissues in comparison to SF/SWNT conduits and negative control groups (figure 14). It could be due to incorporation of FN in the structure of conduits. The nerve bundles ensheathed with myelin and surrounded with matrix consisted of capillaries and connective tissue. Additionally, Schwann cells are usually observed around the axon sheets as dark spots. These cells with elongated nuclei ensheat axons in all parts of peripheral nervous system intimately. It should be mentioned that these finding were confirmed with toluidine blue staining (figure 15). Moreover, the nerve regeneration behavior of the conduits in terms of the number and area of myelinated axons was also investigated (table 2). More myelinated axon area and numbers were observed in SF/SWNT and SF/SWNT/FN conduit groups compared with negative control group, significantly. The high nerve regeneration through the SF/SWNT and SF/SWNT/FN groups may be owing to their porous structure and high permeability and for this they are able to permeate nutrients and exchange metabolites effectively through the tube wall [2], [51]. Additionally, SF/SWNT/FN conduit had statistically more myelinated axon area and numbers than SF/SWNT group. It could be duo to incorporation of FN in the structure of conduits. Some studies reported that fibronectin, laminin and fibrin enhance neurite extension due to adhesion, migration and growth of Schwann cells on the biomaterials [51]–[53]. However, the axon growth in the mid and distal segment of NGCs was very poor (data not shown). Our observation only lasted for 5 weeks, with regeneration nerve recovery incomplete. Totally, it is suggested that the fabricated NGCs could promote and support axon regeneration.

**Table 2:** Statistical analysis of histological assessment after 5

	SF/SWNT/FN	Negative control	SF/SWNT
Number of myelinated axons	684±223	1980±564^a^	3463±1287a,b
Area of myelinated axons	3.78±1.21	7.07±3.55^a^	15.12±2.40a,b

The data represent means and standard deviations.

a *P*<0.05 in comparison to negative control group.

b *P*<0.05 in comparison to negative control and SF/SWNT groups.

#### Immunohistochemistry

**Figure 16:**
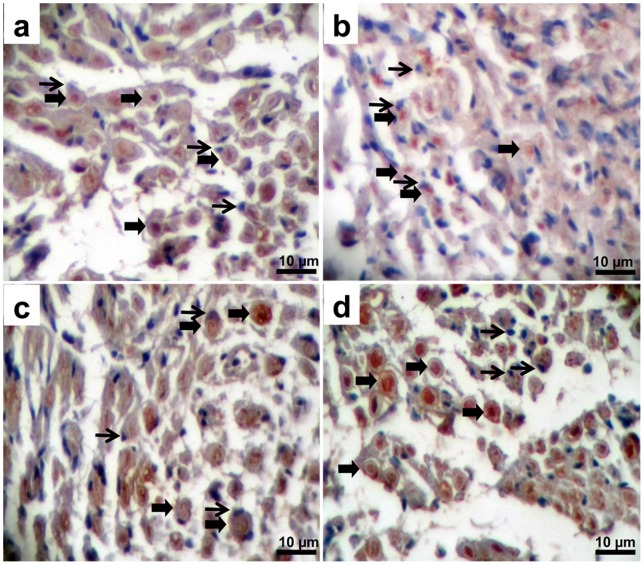
Cross sections of regenerated nerves taken from types of nerve conduits implanted in rats for 5-100. a) Normal nerve, b) Defected sciatic nerve without NGC (control group), c) Defects filled by SF/SWNT conduits, d) Defects filled by SF/SWNT/FN conduits. **Wide arrows**: myelinated axon. **Narrow arrows**: Schwann cells.

Figure 16 displays the S-100 expression of Schwann cells after 5 weeks implantation. Poor regeneration was seen in defected nerve without NGC [control group (figure 16b)]. However, in SF/SWNT conduit modest axonal growth into nerve conduit was notable and a number of Schwann cells were found to be in close contact with axons than control group (figure 16d). Moreover, the S-100 expression of Schwann cells in SF/SWNT/FN conduit was similar to normal nerve (figure 16a and d). It is believed that S-100 expression is responsible for reconstruction and proliferation of Schwann cells in response to peripheral nerve degeneration. So, the expression of S-100 plays an important role in promoting the regeneration of defected nerve [54]. The results concerning immunohistochemistry along with those obtained from histological and electrophysiological assessment confirmed the potential of fabricated conduits in nerve regeneration.

### Conclusion

This study explored a strategy for nerve guide conduit design by introduction and evaluation of topographical, physical and chemical cues. We used advanced analytical tools shifting structural evaluation from bulk properties up to the whole conduit level. Aligned FN-containing nanofibers functioned as topographical cue in the structure of freeze-dried SF/SWNT conduits leading to suitable morphological properties and may open an interesting avenue to use these conduits for *in vivo* nerve regeneration. The bioactivity of FN loaded SF/SWNT conduits was demonstrated by cellular based assay and functioned as a chemical cue to enhance U373 cell lines adhesion and spreading. Nerve conduction velocity was also observed indicating a functional recovery for the injured nerves. Moreover, the histological analyses have also confirmed the appropriate structure of fabricated conduits for nerve regeneration. On the other hand, SF/SWNT/FN conduit had statistically more myelinated axon than SF/SWNT group. The immunohistochemistry evaluation has also confirmed more S-100 expression of Schwann cells in SF/SWNT/FN conduit. In conclusion, our findings pave the way for more generally applicable strategy for biomaterial conduit design. We thus suggest to further explore the potential of these conduits for tissue engineering applications *in vitro* and *in vivo*.
