# Correction: A Biosynthetic Nerve Guide Conduit Based on Silk/SWNT/Fibronectin Nanocomposite for Peripheral Nerve Regeneration

**DOI:** 10.1371/annotation/939b3723-6e52-48ce-9853-e11e368c9f64

**Published:** 2013-10-02

**Authors:** Fatemeh Mottaghitalab, Mehdi Farokhi, Arash Zaminy, Mehrdad Kokabi, Masoud Soleimani, Fereshteh Mirahmadi, Mohammad Ali Shokrgozar, Majid Sadeghizadeh

The affiliation of the third author Arash Zaminy should be "Departemant Of Anatomy and Cell Biology, School of Medicine,Shahid Beheshti University of Medical Sciences, Tehran, Iran" 

